# Nimble adaptations to sexual and reproductive health service provision to adolescents and young people in the early phase of the COVID-19 pandemic

**DOI:** 10.1080/26410397.2024.2372165

**Published:** 2024-07-24

**Authors:** Ahmed K. Ali, Alka Barua, Rajesh Mehta, Venkatraman Chandra-Mouli

**Affiliations:** a Adolescent Sexual and Reproductive Health Consultant, Department of Sexual and Reproductive Health Research which includes the UNDP, UNICEF, UNFPA, WHO and World Bank cosponsored Human Reproduction Programme, Cairo, Egypt. *Correspondence*: aliahme@who.int; b Independent Expert, Goa, India; c Formerly, Regional Adviser, Adolescent Health, WHO South East Asia Regional Office; Currently Independent Expert, New Delhi, India; d Formerly, Scientist, Department of Sexual and Reproductive Health Research which includes the UNDP, UNICEF, UNFPA, WHO and World Bank cosponsored Human Reproduction Programme; Currently Independent Expert, Switzerland

**Keywords:** COVID-19, adolescents, sexual and reproductive health, mental health, service adaptations, digital health

## Abstract

Early in the COVID-19 pandemic, emerging evidence showed that the provision and use of a range of health services, including sexual and reproductive health (SRH) services, were affected. Otherwise, there was little evidence on whether and how they were adapted to maintain the access of different population groups, including adolescents. The study aims to provide an overview of adaptations to adolescent sexual and reproductive health (ASRH) services carried out during the early phases of the pandemic in low- and middle-income countries (LMICs). The Human Reproduction Program (HRP) at the World Health Organization (WHO) called upon WHO and United Nations Populations Fund (UNFPA) regional offices to reach out to organisations that provided ASRH services to submit analytic case studies using a short-form survey. The study team charted information from 36 case studies and performed a content analysis. Results show that the adaptations covered a wide array of SRH services that were provided to a diverse group of adolescents. Most adaptations focused on SRH education and access to contraception in comparison to other SRH services. Over half of the case studies included mental health services, most of which were not provided before the pandemic. The adaptations varied between being face-to-face, remote, digital, and non-digital. Most adaptations complemented a pre-existing service and were nimble, feasible, and acceptable to the targeted adolescents. Lessons learned from this study could be extrapolated into other humanitarian settings and rapid responses for future public health emergencies, provided that rigorous evaluation takes place.

## Résumé

Au début de la pandémie de COVID-19, des données émergentes ont montré que la prestation et l’utilisation d’une gamme de services de santé, notamment de santé sexuelle et reproductive (SSR), avaient été touchées. Par ailleurs, on disposait de peu d’éléments pour déterminer si ces services avaient été adaptés et de quelle manière pour maintenir l’accès de différents groupes de population, y compris les adolescents. L’étude vise à donner un aperçu des adaptations des services de santé sexuelle et reproductive des adolescents réalisées pendant les premières phases de la pandémie dans des pays à revenu faible ou intermédiaire. Le programme de recherche en reproduction humaine (HRP) de l’Organisation mondiale de la santé (OMS) a appelé les bureaux régionaux de l’OMS et du Fonds des Nations Unies pour la population (FNUAP) à encourager les organisations qui assuraient des services de SSR des adolescents à présenter des études de cas analytiques à l’aide d’une enquête en version courte. L’équipe de l’étude a recensé les informations provenant de 36 études de cas et effectué une analyse de leur contenu. Les résultats montrent que les adaptations couvraient un vaste éventail de services de SSR fournis à un groupe diversifié d’adolescents. La plupart des adaptations se sont centrées sur l’éducation en matière de SSR et l’accès à la contraception en comparaison avec d’autres services de SSR. Plus de la moitié des études de cas englobaient les services de santé mentale, dont la plupart n’étaient pas assurés avant la pandémie. Les adaptations variaient entre les services en présentiel, à distance, numériques et non numériques. La majorité des adaptations complétaient un service préexistant et étaient souples, faisables et acceptables pour les adolescents ciblés. Les enseignements tirés de cette étude pourraient être extrapolés à d’autres contextes humanitaires et inspirer des réponses rapides à de futures urgences de santé publique, à condition qu’une évaluation rigoureuse ait lieu.

## Resumen

A principios de la pandemia de COVID-19, la evidencia emergente mostraba que la prestación y el uso de una variedad de servicios de salud, incluidos los servicios de salud sexual y reproductiva (SSR), se vieron afectados. Por lo demás, había poca evidencia de si y cómo se adaptaron esos servicios para mantener el acceso de diferentes grupos poblacionales, entre ellos adolescentes. Este estudio pretende ofrecer una visión general de las adaptaciones a los servicios de salud sexual y reproductiva para adolescentes (SSRA) realizadas durante las fases iniciales de la pandemia en países de bajos y medianos ingresos (PBMI). El Programa de Reproducción Humana (PRH) de la Organización Mundial de la Salud (OMS) instó a las oficinas regionales de la OMS y del Fondo de Población de las Naciones Unidas (UNFPA) a solicitar a las organizaciones que proporcionaban servicios de SSRA que presentaran estudios de casos analíticos utilizando una corta encuesta. El equipo del estudio registró la información de 36 casos estudiados y realizó el análisis de contenido. Los resultados muestran que las adaptaciones abarcaron una gran variedad de servicios de SSR proporcionados a un grupo diverso de adolescentes. La mayoría de las adaptaciones se centraron en la educación sobre SSR y en el acceso a métodos anticonceptivos, en comparación con otros servicios de SSR. Más de la mitad de los casos estudiados comprendían servicios de salud mental, la mayoría de los cuales no se proporcionaban antes de la pandemia. Las adaptaciones variaron entre ser presenciales, remotas, digitales y no digitales. La mayoría de las adaptaciones suplementaban un servicio preexistente y eran ingeniosas, factibles y aceptables a la adolescencia objetivo. Las lecciones aprendidas de este estudio podrían extrapolarse a otras situaciones de crisis humanitarias y respuestas rápidas a futuras emergencias de salud pública, siempre y cuando se realice una evaluación rigurosa.

## Introduction

Adolescents and young people constitute a large part of the world’s population, estimated at 1.8 billion, of which 1.3 billion are aged 10–19, and around 90% live in low- and middle-income countries (LMICs).^1^ Since the International Conference on Population and Development (ICPD) in 1994, there has been global progress in many areas of adolescent sexual and reproductive health (ASRH).^2,3^ However, evidence from previous public health emergencies showed that adolescent health, including sexual and reproductive health (SRH), tended to be neglected,^4–6^ and their access to SRH services was impacted, especially when resources were diverted to containment responses.^6,7^ Early in the course of the COVID-19 pandemic, according to a scoping review of evidence gathered in February 2021, access of adolescents to SRH services was affected, specifically access to contraception, menstrual health products, and anti-retroviral treatments (ARTs).^8^ While the exact reasons remain to be studied, many of the contributing factors included mobility restrictions, limited in-person care, shortages in medical supplies and SRH commodities,* the refusal of healthcare workers to provide services, and the closure of youth-friendly services, community centres, and schools.^8,10–14^ Furthermore, there have been reports that the challenges to access were more pronounced among underserved adolescents.^15^

As the international community became aware of the challenges that the pandemic might impose on access to services, several guidelines were issued to modify service delivery to mitigate restrictions enforced by the effects of the pandemic.^16^ For instance, in May and June 2020, the WHO published two documents about maintaining essential health services and community-based care in the context of the COVID-19 pandemic,^17,18^ and UNFPA published a supplemental technical brief titled *Not on Pause: Responding to the sexual and reproductive health needs of adolescents in the context of the COVID-19 crisis.*^19^ This technical brief set out recommended actions for the delivery of services, monitoring, and considerations for the transition towards restoration and recovery for each component of the Guttmacher-Lancet Commission’s essential package of sexual and reproductive health and rights (SRHR) interventions for adolescents, in the context of COVID-19.

While service providers worked during the early phases of the pandemic to adapt SRH services for adolescents, at the time of this study, there was limited research on which SRH services were delivered during the pandemic, to which groups of adolescents, and how these programmes were adapted to maintain access of adolescents to SRH services. Our paper expands on the reports of initiatives from the Philippines, Zimbabwe, South Africa, and other countries in adapting SRH services delivered to adolescents^20–22^ in terms of geographical scope, types of SRH services, and the groups of adolescents reached through nimble adaptations to SRH services by organisations during the early phases of the pandemic in LMICs. Our research question is: How did organisations from LMICs respond to the different SRH needs of adolescents during the early phase of the COVID-19 pandemic?

## Methods

### Data collection tool

The study team developed a simple analytic case study tool using a survey that included questions that were categorised into four sections. The first was a background section that explored the organisation's mandate and the SRH services that it provided to adolescents before the pandemic. The second section explored the adaptations made to the SRH services that were provided by the organisation to adolescents during the early phase of the pandemic. The third section explored the reasons behind carrying out these adaptations and how they were developed. The final section explored whether the adaptations were monitored.

### Data collection procedures

The study team gathered case studies of adaptations by organisations to provide SRH services to adolescents in line with the recommendations made in the UNFPA technical brief.^19^ To gather these case studies, the HRP/WHO called upon WHO and UNFPA regional offices to identify organisations to submit case studies. Eligible responses had to satisfy two conditions: (i) the submitting organisation had to be from an LMIC and (ii) completeness and adherence to the tool format. There were no limits on the number of submitting organisations per country or region, the type of SRH service provided, or the targeted group of adolescents. Two independent researchers contacted the organisations, provided them with the study survey, and followed up with them to ensure proper reporting and completeness**.** As the pandemic rolled out differently in different countries, it was important to provide enough time for data collection to gather information about a wider variety of adaptations to SRH services in different contexts. Data collection started in April 2020 and was completed by December 2020. While the data collection period coincided with two waves of the COVID-19 pandemic,^23^ particularly in Asia^24^ and Africa,^25^ the information shared was mostly specific to the first wave or directly after. Out of 40 case studies gathered by the team, three were excluded due to their incompleteness, and one was excluded due to a lack of adherence to the predetermined format.

### Data abstraction

The study team abstracted the information using an abstraction sheet that included domains about characteristics of the organisations, types of SRH services provided before and during the early phase of the pandemic, adolescents and young people targeted by these services, adaptations used by these organisations to deliver these services, and whether they were face-to-face, remote, digital, or non-digital, in addition to domains about the design, development, and monitoring of these adapted services during the early phase of the pandemic.

### Data analysis and synthesis

Two researchers were involved in the analysis of the data and the synthesis of the results. They developed a framework of four sub-questions that were aligned with the research question to inform the data analysis and synthesis. These questions were: (1) Which SRH services were provided by the organisations in LMICs to adolescents during the pandemic? (2) Which adolescent groups were targeted by these services? (3) What were the adaptations used by the organisations to provide the SRH services to these adolescents? and (4) How were these adaptations developed, delivered, and monitored? The study team charted the data, reviewed the abstracted information, then performed a content analysis and a narrative synthesis of the abstracted data and presented the study findings in correspondence to the mentioned framework.

### Supplementary  data

The full set of case studies is available in the Medicus Mundi Bulletin entitled: Lessons learned from nimble adaptations to organisations’ responses to the sexual and reproductive health (SRH) needs of adolescents in the context of the COVID-19 crisis. https://www.medicusmundi.ch/de/wissen-und-lernen/reflexions-und-lernformen/who-case-studies/.

### Ethics statement

Confirmation that this study was exempt from ethics committee approval was obtained before data collection began from the Research Protocol Review Panel of the World Health Organization’s Department of Reproductive Health and Research, based on a standing exemption on the grounds that this was a public health activity that drew out managerial information from the service managers, plans and reports in the public arena and did not involve primary data collection from either the providers or the beneficiaries of such programmes.

## Results

We provide an overview of the characteristics of the organisations, SRH programmes, and target audiences involved in the 36 case studies, in addition to the development, delivery, and monitoring of the adaptations used by the organisations to provide SRH services to adolescents and young people during the early phase of the pandemic.

### Case studies’ characteristics

#### Regions, countries, and organisations

The 36 case studies were from four different world regions, 16 countries, and 41 organisations. In many instances, there were multiple case studies per country, but each case study was unique and came from a different submitting organisation. Of these 36 case studies, 17 were from Africa, 16 were from Asia, one was from the Pacific region, and one was from Latin America. In addition, there was a global case study submitted by the International Planned Parenthood Federation (IPPF) that presented examples from its member associations. In Africa, the 17 case studies were from nine countries: Benin (*n* = 1), the Democratic Republic of Congo (DRC) (*n* = 1), Kenya (*n* = 2), Malawi (*n* = 1), Namibia (*n* = 1), Nigeria (*n* = 4), South Africa (*n* = 1), Uganda (*n* = 3), and Zimbabwe (*n* = 3). In Asia, the case studies were from five countries: India (*n* = 9), Laos (*n* = 1), Myanmar (*n* = 4), Nepal (*n* = 1), and the Philippines (*n* = 1). The three remaining case studies were from the Pacific region (Fiji), Latin America (Argentina), and finally, the global case study submitted by IPPF. In four of the case studies, responses were submitted by multiple organisations that collaborated in each case. Therefore, the total number of organisations (*n* = 41) is higher than the total number of case studies (*n* = 36). The submitting organisations were mostly non-governmental organisations (*n* = 29), in addition to United Nations agencies (*n* = 5), governmental sectors (*n* = 4), for-profit organisations (*n* = 2), and one academic institution. Detailed results appear in Table 1.

**Table 1.** Characteristics of 36 case studies on adoptation to adolescent SRH services during COVID-19 Pandemic

Number of case studies per region	Number of case studies per country	Organisation	Type of organisation	Primary SRH service(s)
Africa (*n* = 17)	Benin (*n* = 1)	OneWorld Benin	Non-Governmental Organisation	SRH education
	The Democratic Republic of Congo (*n* = 1)	Ailes du Coeur	Non-Governmental Organisation	Contraception
	Kenya (*n* = 2)	University of Nairobi	Academic Institution	HIV care, LGBTQ + support
		Youth in Action (Y-ACT) / Amref Health Africa	Non-Governmental Organisation	SRH commodities
	Malawi (*n* = 1)	Safeguard Young People (SYP) / UNFPA ESARO	UN agency	SRH education
	Namibia (*n* = 1)	Namibia Planned Parenthood Association (NAPPA)	Non-Governmental Organisation	SRH education (CSE) and contraception
	Nigeria (*n* = 4)	Girls Ladies and Women Empowerment Initiative (GLW)	Non-Governmental Organisation	SRH education, menstrual health, and SGBV care
		Society for Family Health (SFH)	Non-Governmental Organisation	Contraception
		Stand To End Rape Initiative (STER)	Non-Governmental Organisation	SGBV care
		Stand With A Girl (SWAG)	Non-Governmental Organisation	SRH education
	South Africa (*n* = 1)	Footprints Foundation	Non-Governmental Organisation	Menstrual health
	Uganda (*n* = 3)	Marie Stopes International-Uganda	Non-Governmental Organisation	Contraception
		UNFPA and Jumia	UN agency and private sector	SRH commodities
		UNFPA and SafeBoda	UN agency and private sector	SRH commodities
	Zimbabwe (*n* = 3)	Africaid (Zavandiri) and the Ministry of Health and Child Care in Zimbabwe	Non-Governmental Organisation and government	HIV care
		Ministry of Education, UNFPA, and UNESCO	UN agencies and government	SRH education
		Students and Youth Working on Reproductive Health Action Team (SAYWHAT)	Non-Governmental Organisation	SRH education
Asia (16)	India (*n* = 9)	Centre for Catalysing Change (C3)	Non-Governmental Organisation	SRH education and menstrual health
		Family Planning Association of India (FPAI)	Non-Governmental Organisation	SRH education (CSE), menstrual health, and ASRH services
		Foundation for Reproductive Health Services-India (FRHSI)	Non-Governmental Organisation	Contraception and safe abortion care
		Human Touch Foundation	Non-Governmental Organisation	HIV care
		Institute of Health Management, Pachod (IHMP)	Non-Governmental Organisation	SRH education, contraception, maternal health and SGBV care
		Love Matters	Non-Governmental Organisation	SRH education
		MAMTA Health Institute for Mother and Child	Non-Governmental Organisation	Providing contraceptive information and services
		Society for Nutrition, Education and Health Action (SNEHA)	Non-Governmental Organisation	SRH education and menstrual health
		Talking About Reproductive and Sexual Health Issues (TARSHI)	Non-Governmental Organisation	SRH education
	Laos (*n* = 1)	Ministry of Health in Laos	Government	Human papillomavirus vaccination
	Myanmar (*n* = 4)	BBC Media Action	Non-Governmental Organisation	SRH education
		Burnet Institute	Non-Governmental Organisation	SRH education and menstrual health
		Marie Stopes International-Myanmar (MS-M)	Non-Governmental Organisation	Contraception and SRH education
		Myanmar Medical Association (MMA)	Non-Governmental Organisation	SRH education (CSE)
	Nepal (*n* = 1)	Beyond Beijing Committee (BBC)	Non-Governmental Organisation	SRH education, safe motherhood and menstrual health
	Philippines (*n* = 1)	Family Planning Association of the Philippines (FPOP)	Non-Governmental Organisation	SRH education (CSE), SGBV care, HIV testing, contraception, and safe post-abortion care
Latin America (1)	Argentina (*n* = 1)	Directorate of Adolescents and Youth and the Ministry of Health (DiAJu)	Government	SRH education, safe abortion care and SGBV care
Pacific (1)	Fiji (*n* = 1)	Reproductive Family Health Association (RFHAF)	Non-Governmental Organisation	Contraception
Global (1)	Multiple countries (*n* = 1)	International Planned Parenthood Federation (IPPF)	Non-Governmental Organisation	SRH education (CSE), contraception and SGBV care

### Adaptations to SRH programmes delivered to adolescents during the COVID-19 pandemic

#### SRH programmes

The case studies covered adaptations to multiple SRH programmes that were provided to adolescents during the early phase of the pandemic. The majority of these programmes included: (i) SRH education, including comprehensive sexuality education (CSE)† programmes; (ii) access and referral to contraception services; (iii) access and referral to HIV care; (iv) menstrual health education; (v) access to safe abortion and/or post-abortion care; (vi) access and referral to sexual and gender-based violence (SGBV) care; and (vii) distribution of SRH commodities. In addition, there was one case study that discussed adaptations to an HPV vaccination programme during the pandemic. The majority of the case studies discussed adaptations to SRH education programmes which included 22 case studies, of which five were CSE adaptations. Out of the 36 case studies, 16 reported adaptations related to multiple SRH services that were undertaken concurrently. Detailed results appear in Table 1.

#### Target audiences

The majority of the adaptations targeted adolescent girls, adolescent school students, and adolescents in nearby communities, and to a lesser extent, out-of-school adolescents, and adolescents living with HIV. The groups least mentioned were pregnant or married adolescents, adolescents living in rural or urban informal settings, adolescents with disabilities, and lesbian, gay, bisexual, transgender, queer, or other (LGBTQ+) adolescents. Detailed results appear in Table 2.

**Table 2.** Adaptations to adolescent SRH services during the COVID-19 pandemic

Organisation	Adaptations	Target audience	Innovative actions	Monitoring and follow-up
Africaid (Zavandiri) and the Ministry of Health and Child Care- Zimbabwe	Service delivery	Adolescents living with HIV	Established a virtual case management system and provided virtual clinical and psychosocial support services, in addition to treatment reminders	Virtual case management data; services and referral data; follow-up on health facilities; and feedback from staff and beneficiaries, in addition to peer advisory boards
	• Established a virtual case management system for adolescents living with HIV			
	• Built virtual support groups and provided virtual clinical and psychosocial HIV services			
	• Community Adolescent Treatment Supporters (CATS) conducted follow-up home visits, distributed ARTs and provided counselling and support and measurement of viral load			
	• CATS provided reminders via the Zvandiri Mobile Database Application (ZVAMODA) for adherence to treatment			
Ailes du Coeur - Democratic Republic of Congo	Service promotion, delivery and referrals	Adolescents	Established mechanical dispensers to distribute condoms and provided a dedicated WhatsApp messaging service to receive requests for information, contraceptives, and clinic appointments, and to coordinate referral and scheduling of services	Overall reach and number of commodities distributed
	• Provided contraceptives to young people through their clinics while maintaining COVID-19 safety protocols			
	• Installed mechanical dispensers to provide condoms without human interaction and added masks and gloves next to the dispensers			
	• Disseminated a WhatsApp number to coordinate young people’s requests for information, access to certain contraceptives, and scheduling of appointments			
	Community outreach			
	• Assigned young people as focal points and contraceptive suppliers who distributed contraceptives – door to door- to adolescents and young people in their neighbourhoods			
BBC Media Action - Myanmar	ASRH education	Adolescents with a specific focus on adolescents with disabilities	Provided SRH education through digital means; radio, animation, and quizzes through their Facebook page	Social media metrics that included reach and engagement data, in addition to follow-up reports
	• Supported the development of radio and digital content by Myanmar Independent Living Initiative (MILI) for adolescents with disabilities			
	• Carried out ASRH education activities through social media using interactive quizzes and personalised messages and provided responses to queries and updated their Facebook page ASRH content using motion graphics and animations			
BBC – Nepal	ASRH education	Adolescent girls	Promoted SGBV and psychosocial support hotlines, set up a toll-free number for adolescents counselling on safe motherhood, and provided ASRH education to adolescent girls via digital platforms with focus on MH	Number of calls and services provided, in addition to number of commodities distributed
	• Shared information to adolescent girls via digital platforms about menstrual health (MH) and how to make reusable napkins at home during the lockdown			
	Service promotion, delivery and referrals			
	• Distributed menstrual products and food packages			
	• Set up a 24-hour toll-free number -in collaboration with partner organisations- to provide counselling services by doctors and midwives on safe motherhood and reproductive health			
	• Promoted and referred adolescents to services available by the government and partner organisations, such as SGBV hotline and psychosocial counselling and support services			
Burnet Institute - Myanmar	ASRH education	School students	Provided SRH education to students via distance learning sessions via Zoom, Google Meet, and Facebook messenger	Follow-up calls, virtual meetings and home visits were used to follow-up on students’ learning progress and parents’ engagement
	• Adapted their Integrated Multi-Sectoral Approach (IMSA) programme in schools to be add an online ASRH earning component via Zoom, Google Meet, and Facebook messenger			
	• Continued in-person group ASRH education sessions, in small groups to engage parents and guardians, teachers, and young people			
	Community outreach			
	• Continued to distribute commodities such as: menstrual hygiene kits, face masks, food packages, among others			
Centre for Catalysing Change (C3) – India	ASRH education	Adolescents	Provided SRH education through social media using video and audio messages, referred adolescents to hotlines providing counselling services	Follow-up data integrated at the National Adolescent Health (RKSK) Program platform, in addition to monthly program reviews and follow-up phone calls
	• Peer educators disseminated ASRH and COVID-19 information via ten video and audio messages distributed via social media and WhatsApp			
	Community outreach			
	• Distributed sanitary napkins, and folic acid tablets and referred adolescents to available services such as counselling hotlines			
Directorate of Adolescents and Youth and the Ministry of Health (DiAJu) - Argentina	ASRH education	School students	Provided SRH education through social media, phone calls and radio messages, provided virtual counselling, phone and teleconsultations, and provided referrals for SGBV care and abortion service to appropriate agencies	Follow-up reports; stakeholders’ interviews; feedback from services’ users; in addition to reach, coverage, and utilisation data
	• Provided SRH messages to adolescents through social media, phone calls, and local radio			
	Service delivery and referrals			
	• Established an intersectoral network of partners that covered sectors such as education, justice, social development, and other local institutions for coordinated responses			
	• Provided virtual counselling, phone consultations and teleconsultations – after developing appropriate guidelines, to adolescents			
	• Referred cases of suspected sexual abuse, suicide attempts, domestic or gender-based violence, and requests for abortion counselling and services, to rights protection agencies, territorial focal teams and other agencies			
Family Planning Association of India (FPAI) – India	Service delivery	In and out of school adolescents	Provided teleconsultations, online CSE sessions, and distributed biodegradable sanitary napkins*	Follow-up surveys; observations; and feedback from adolescents
	• Provided adolescents with teleconsultations through Zoom and WhatsApp about SRH and COVID-19-related issues			
	ASRH education			
	• Adapted the delivery of their CSE programme to an online mode			
	• Engaged with students through social medial and carried out virtual activities and competitions such as a poetry competition on menstruation and a mobile phone film-making competition			
	Community outreach			
	• Distributed biodegradable sanitary napkins to adolescents			
FPOP – Philippines	ASRH education	Adolescents	Developed a virtual safe space for young people and set up hotlines for the provision of SRH information, counselling and services including post-abortion care, and SGBV care, in addition they delivered SRH commodities, door to door, and maintained their CSE online sessions	Social media metrics, including ‘reach’
	• Maintained online CSE sessions and shared messages on SRH via social media			
	Service delivery and referrals			
	• Launched the YouRHotline, a Facebook-based platform to create a virtual community and safe space for adolescents where professionals respond to young people on their SRH (including SV and IPV) and mental health queries and concerns			
	• Set up additional hotlines for post-abortion care, and SGBV referrals			
	• Referred adolescents in need to their clinics, and when needed, their partners			
	• Offered counselling and psychological services as part of SGBV care			
	Community outreach			
	• Delivered SRH commodities door to door such as HIV- and pregnancy-testing tests, and contraceptives			
	• When implants were requested, a nurse or a midwives took on the visit			
Footprints Foundation – South Africa	Community outreach	Adolescent girls	Delivered menstrual health products to adolescents*	Overall reach and number of distributed commodities
	• Delivered menstrual health products to ten schools and adolescents in need at shelters, facilities for persons with disabilities, and child and youth care centres			
Foundation for Reproductive Health Services-India (FRHSI) – India	Service promotion and delivery	Adolescent girls	Provided companionship and facilitated transportation to mitigate restrictions imposed by COVID-19*	Field visits; observation; video reflective accounts; online meetings; in addition to the use of reach, coverage and service utilisation data
	• Provided contraception and safe abortion services in compliance with COVID-19 safety protocols with modified services delivery guidelines			
	• Promoted contraception and safe abortion services to young women and adolescent girls through clinical health outreach teams and trained accredited social health activists (ASHAs)			
	• ASHAs accompanied adolescent girls and young women to places of service delivery to navigate mobility restrictions during the lockdown period			
	• Ambulances were provided to pick up and drop clients as the organisations ambulances had the permission to move around even where other vehicles were not			
	• Some services were subsidised			
	Community outreach			
	• ASHAs and clinical health outreach teams counselled women about available services and helped them make decisions regarding an unwanted pregnancy			
Girls Ladies and Women Empowerment Initiative (GLW) – Nigeria	ASRH education	Adolescent girls	Used social media and radio edutainment programmes for ASRH education, and distributed packages to adolescent girls and their households	Feedback from youth champions in selected communities; input from radio interactions; and social media ‘engagement’ metrics
	• Used social media, radio and edutainment programmes to provide information on menstrual health, gender-based violence, other sexual reproductive health issues, and mental health, in addition to COVID-19 prevention			
	Community outreach			
	• Distributed packages that included soaps, hand sanitisers, face masks, and sanitary pads.			
	• Engaged community leaders and health professionals to contribute to sensitisation activities and correct myths and misconceptions about COVID-19			
Human Touch Foundation – India	Service delivery	Adolescents	Provided online psychosocial support and tele-counselling to promote adherence to the ARTs, and provided ART supplies at home, and commodities.	Monthly tracking of the number of distributed ART supplies and commodities, in addition to the use of ‘reach’ and service utilisation data
	• Provided Anti-Retroviral Therapy (ART) supplies to children and adolescents			
	• Provided online psychosocial support and tele-counselling to promote adherence to the ARTs during the pandemic			
	Community outreach			
	• Distributed commodities such as basic food grains, groceries, antiseptic soaps, and masks			
Institute of Health Management, Pachod (IHMP) - India	ASRH Education	Adolescent girls, out of school adolescents, adolescents living in rural areas, and married adolescents	Not applicable	Not applicable
	• Continued their community-based life skills education, peer education, girls’ collectives’ activities, and male- engagement sessions, while maintaining COVID-19 safety requirements			
	Service delivery			
	• Provided outreach clinics that provided maternal and neonatal services, contraception, and other reproductive health services to married adolescent girls or referred them to health centres that continued to provide the services			
	• Operated safe spaces for students, and out-of-school girls, in rural and urban areas			
	• Provided counselling for maternal health care and contraception to married adolescents			
	Community outreach			
	• Followed up on adolescent girls’ health with monthly health and morbidity assessments implemented by ASHAs			
International Planned Parenthood Federation (IPPF)	ASRH education	Adolescents	IPPF MAs developed synchronous and asynchronous online SE resources and provided online counselling	Follow-up survey, ‘reach’ data; and users’ feedback
	• Most IPPF Member Associations (MAs) adapted their in-person sexuality education resources for digital use and used synchronous online platforms such as Zoom			
	• Some MAs conducted individual education sessions, while others used small group sessions			
	• Some MAs in the countries which were not immediately affected by the pandemic continued their school-based SRH education programmes while complying to COVID-19 safety protocols			
	Service delivery			
	• Facilitated the access of young people to online ASRH counselling such as contraception, and SGBV			
Love Matters India (LMI) – India	ASRH education	Adolescents	Through their online platform, LMI provided weekly radio programme and introduced a ‘find a clinic’ feature, and a WhatsApp messaging service to engage with young people and respond to their queries	Focus group discussions; and ‘reach’ data
	• LM’s radio episodes continued to reach out over 500,000 young people with SRH information weekly			
	• Launched the Love Matters India (LMI) WhatsApp service to engage with young people and respond to their queries			
	Service promotion and referrals			
	• Launched the “Find a Clinic Feature,” which listed clinics where young people could access SRHR/FP services			
MAMTA Health Institute for Mother and Child- India	ASRH education	Adolescents	Utilised word of mouth endorsements to promote family planning and installed kiosks to provide SRH information and contraceptives, in addition to condom boxes for no-contact access in the hospitals*	Follow-up surveys; ‘reach’ and service utilisation data
	• Provided family planning messages through positive gossiping by mothers in law “Chugal Chachi” about the positive impact of adopting family planning methods			
	Service delivery			
	• Female health workers operated through kiosks that were installed nearby the outpatient departments of hospitals where they disseminated information about contraception methods, and distributed contraceptives to those interested			
	• Placed condom boxes in hospitals as a place for a quick grab-and-go			
Marie Stopes International-Myanmar	ASRH Education	Adolescents	Provided family planning teleconsultation services and online counselling in addition to online SRHR talks via social media or virtual meetings apps	Service utilisation data; in addition to direct follow-up meetings
	• Modified their youth corner SRHR talks to be provided through online platforms via social media or virtual meetings apps			
	• Maintained their CSE activities but with reduced-size classes			
	Service promotion and delivery			
	• Promoted the availability of their services via social media			
	• Continued operating their family planning clinics in 12 states after introducing an appointment booking systems to reduce waiting time, and to ensure compliance to COVID-19 safety protocols			
	• Included teleconsultation services for in addition to online counselling for interested young clients			
Marie Stopes International-Uganda	Service promotion, delivery and referrals	Adolescents	Adapted the ‘refer a friend system’ by using poster and flyers to promote the availability of clinics during the pandemic*	Part of a randomised trial; process level indicators; data collection in progress**
	• Adapted their ‘refer a friend’ system by using posters and flyers distributed by peer educators and village health mobilisers to engage/retain adolescent clients following up at their ‘BlueStar clinics’			
	• Promoted their SRH services by SMS with images and texts that appeal to young people			
	• Provided adolescents with contraceptive counselling and SRH education at the clinics while complying to COVID-19 safety protocols			
Ministry of Education, UNFPA, and UNESCO- Zimbabwe	ASRH Education	In and out of school adolescents	Developed and conducted nationwide radio CSE lessons	Overall reach and satisfaction of adolescent listeners
	• Provided nationwide radio lessons as part of the Guidance and Counselling and Life Skills Education (G&C-LSE); the local CSE programme and covered topics such as health and well-being, relationships, children’s rights, safety and protection and GBV			
Ministry of Health- Laos PDR	Service promotion and delivery	Adolescent girls	Maintained HPV vaccination despite schools’ closure through the integration of vaccination in health centres services and outreach activities*	Reach, and vaccination data
	• Human Papilloma Virus (HPV) vaccinations were shifted from schools due to school closures and became integrated in the Ministry of Health in Laos’ wider activities			
	• The National Immunization Programme (NIP) and the Maternal and Child Health Center (MCHC) invited all eligible girls to visit nearby health centres or to attend outreach sessions where HPV vaccination is provided			
	• Messages were sent through various channels to reach communities, parents, and girls to encourage them to visit health centres or to check with village health volunteers and village heads if there would be HPV mop-up/outreach sessions to receive HPV vaccinations			
Myanmar Medical Association (MMA) - Myanmar	ASRH Education	Adolescents	Provided SRH education through social media and virtual meeting apps	Knowledge assessment; reach and coverage data; feedback from users’ comments on the Facebook page
	• Provided SRH education to adolescents through their Facebook based project; ‘Knowledge-to-your-finger-tip’ and online ASRH Zoom-based trainings that employed different approaches and learning aids such as presentations, videos, songs and case scenarios			
Namibia Planned Parenthood Association (NAPPA) -Namibia	ASRH Education	In and out of school adolescents	Used a dedicated WhatsApp messaging service to provide COVID-19 and ASRH information	Community-led monitoring: reach, coverage and services’ utilisation
	• Disseminated messages on SRH, GBV, and COVID-19, and engaged young people through peer educators and WhatsApp messaging			
	Service delivery and referrals			
	• Provided mental health support through door-to-door outreach and mobile vans			
	• Followed up on adolescents’ well-being with health screenings and coordinated referrals to NAPPA family planning clinics and youth centres			
OneWorld Benin	ASRH Education	Adolescents	Provided ASRH education through social media, mobile app, and online platform	‘Reach’ data
	• Continued their ASRH education through their mobile app and online platform			
	• Added a social media component with visual messages, and organised debates to engage with young people and raise their awareness on GBV, parent-to-child communication, menstrual health, women’s rights, early and unintended pregnancy as well as HIV and STIs			
Reproductive Family Health Association (RFHAF) – Fiji	Service promotion and delivery	Adolescents	Provided family planning counselling through their newly established helpline	Surveys; monthly meetings; observations; social media feedback
	• Promoted the availability of family planning services on social media channels			
	• Provided family planning counselling services by establishing a helpline -in partnership with the Ministry of Women in Fiji			
	• Mobilised its pool of nurse midwives, counsellors, and youth volunteers to reach communities and young people with SRH information and services via mobile clinics and aided in booking appointments with clinic staff			
	• Static clinics’ opening hours were extended and COVID-19 safety protocols were followed			
Safeguard Young People (SYP)/UNFPA ESARO – multiple countries	ASRH education	Adolescents	Integrated ASRH ‘Tune ME’ website as a part of basic internet consumption bundles thus do not require additional costs	Website data analytics; page visits and reach
	• Provided personalised and reliable information on COVID-19 and SRHR topics; reproductive health, menstrual health, and gender-based violence on ‘TuneMe’ website and social media, and was provided as part of the basic internet bundle			
Society for Family Health (SFH)/A360 – Nigeria	ASRH education	Adolescent girls	Utilised social media; Facebook page, and mass messaging (SMS) to promote their services and refer adolescents to available clinics	Social media metrics that included ‘reach’, in addition to feedback from adolescent girls and healthcare providers
	• Sent mass messages (SMS) – at state level- to provide information bites and interactive myth-buster quizzes to engage with adolescent girls on topics such as COVID-19, SRH, gender-based violence, and livelihood development			
	Service promotion, delivery and referrals			
	• Partnered with the Ministry of Health to maintain SRH care as part of the essential services			
	• Launched an online campaign using a Facebook page to promote family planning and refer adolescent girls to family planning clinics			
	• Provided adolescent girls with contact numbers of youth-friendly providers who would arrange for them to visit a facility to receive contraceptives			
	Community outreach			
	• Youth mobilisers and community health workers engaged with adolescent girls living in their communities to facilitate access to DMPA-SC contraceptives and follow-up on injections			
Society for Nutrition, Education and Health Action (SNEHA) – India	SRH education	Adolescents living in urban informal settings	Provided SRH education via phone and WhatsApp messaging service, provided mental health screening and counselling by phone, and Distributed menstrual health products	Reach and services utilisation data
	• Under the Empowerment, Health, and Sexuality of Adolescents (EHSAS) initiative, group education sessions were conducted for both boys and girls on SRH including menstrual health, through WhatsApp group video calls			
	Service delivery			
	• Developed a screening tool to identify adolescents who need psychosocial support and provided them with counselling over the phone			
	Community outreach			
	• Male adolescents delivered sanitary napkins and other MH products to households in need			
Stand To End Rape Initiative (STER) – Nigeria	Service delivery and referrals	Adolescent girls	Used phone services, text messaging and virtual meeting apps to provide mental health support, counselling and proper referrals for those who experienced GBV	Feedback from service users, and a real-time follow-up data matrix for referral and management
	• Provided virtual support using phone services to support those who experienced GBV and link them to appropriate service providers			
	• Provided mental health counselling via Zoom and Skype while adopting text message counselling for clients that preferred that method			
	• Arranged for transportation when needed, and facilitated scheduling of services			
	Community outreach			
	• Carried out community engagement activities to sensitise people about the likelihood of an increase in GBV and inform them on how to prevent and manage GBV during the outbreak			
Stand With A Girl (SWAG) – Nigeria	ASRH education	Adolescent girls	Provided SRH education using WhatsApp messaging service via voice messages and group discussions	Knowledge assessment; and feedback from adolescent girls regarding selected topics
	• Created a virtual safe space to discuss with adolescent girls ASRH topics, as they provided virtual presentations via voice messages using WhatsApp and followed up with discussions on topics such as GBV, menstruation, female genital mutilation (FGM), contraceptives, leadership, communication, self-esteem, advocacy, storytelling, and public speaking			
Students and Youth Working on Reproductive Health Action Team (SAYWHAT) – Zimbabwe	ASRH education	School students	Provided ASRH education through social media and virtual meeting programs, and strengthened their call centre to provide counselling services, psychosocial support and referrals for SGBV services	Reach data; social media metrics and app data analytics; monthly reports of activities; and analysis of call centre queries
	• Held discussions on different SRH issues on social media platforms such as Facebook, WhatsApp, Zoom, and Twitter			
	• Contributed to video and radio talk shows– which were broadcasted on social media platforms			
	Service delivery and referrals			
	• Strengthened their call centre to ensure that young people who reached out could receive online counselling services, psychosocial support and referrals for services that address GBV			
	• Offered online referrals for SRH services			
Talking About Reproductive and Sexual Health Issues (TARSHI)- India	ASRH education	School students	Developed an online ASRH resource, and continued their online ASRH courses and sessions with young people	Reach data; and feedback from online resources’ users
	• Created an online web-based resource ‘SRHR in COVID Times’ for young people, researchers and journalists that explored the interrelationship between COVID-19, SRHR and adolescent well-being			
	• Provided online SRHR courses and sessions for adolescents and young people			
	• Conducted several sessions for parents on talking to their children about sexuality-related issues and on SRHR for students in college			
UNFPA and Jumia-Uganda	ASRH education	Adolescents	Partnered with an e-shop app (Jumia) to ensure adolescents’ accessibility to SRH products and referral of adolescent clients in need to MSI hotline	Jumia app data analytics including reach and distribution data
	• Customer service employees provided ASRH information			
	Service delivery and referrals			
	• Expanded Jumia’s SRH products inventory to include maternity kits, HIV testing kits, pregnancy testing kits, condoms, and menstrual hygiene products, with support from UNFPA.			
	• Enabled clients to order reproductive health commodities online and orders were exempted from delivery fees			
	• Customer service employees referred those who needed further support to MSI hotline			
UNFPA and SafeBoda – Uganda	Service delivery	Adolescents	Utilised motorcycle taxi app (SafeBoda) and created an e-Pharmacy for purchase and ‘free’ delivery of SRH products	SafeBoda app data analytics including reach and distribution data
	• Developed with SafeBoda – a motorcycle taxi app- an e-Pharmacy titled “Personal Health Pharmacy” to enable clients to order reproductive health commodities online and onboarded 10 pharmacies with the support of MSI, Population Services International (PSI), AfriPads, and Holic Pads for the initiative			
	• Delivered SRH products free of charge, including the delivery of free condoms provided by the government			
	• Provided free condoms to community health agents, including peer educators and village health teams to distribute in their communities.			
University of Nairobi-Kenya	Service promotion and delivery	Adolescents living with HIV, LGBTQ+ adolescents, pregnant adolescents, and adolescent parents	Developed online mental health training, psychosocial support groups and online counselling	Developed process and impact indicators; status of data collection is unclear
	• Established virtual groups through Zoom and Skype to provide mental health services, primarily for pregnant adolescents and adolescent parents which included self-care, psychological first aid, and referral pathways to relevant services			
	• Developed a web-based counselling and psychosocial support services for LGBTQ+ adolescents			
	• Youth leaders, health workers and community organisations promoted these services			
Youth in Action (Y-ACT)/AMREF Health Africa – Kenya	Provision of commodities	Adolescents	Providing commodities in partnership with other organisations*	Overall reach and number of distributed commodities
	• Partnered with community-based organisations to support the distribution of sanitary pads and food packages to girls in households or safe houses, or to homeless families.			

* Innovations that did not take on a digital form.

** At the time of conducting this study.

### Development of adaptations to the SRH programmes

During the early phase of the pandemic, particularly its first wave, organisations reported that governments issued lockdown measures that included mobility restrictions or curfews and the avoidance of gatherings. Many public and private health services were completely or partially shut down. Similarly, schools and recreational facilities for young people were closed, and their services were discontinued. In many instances, adolescents and healthcare providers were impacted by the mobility restrictions, thus limiting access to SRH services. Therefore, organisations developed their adaptations primarily to mitigate the lack of access to SRH services due to the pandemic containment measures using remote, mostly digital approaches. The lockdown measures were imposed during different periods across the countries as the pandemic waves peaked and waned differently. In areas where lockdown measures were not yet imposed or did not mandate services’ closure, in-person services were either adapted to be provided in compliance with COVID-19 safety protocols, or as a hybrid of in-person and remote methods. In some countries, mostly in Africa, the first wave of the pandemic was less severe in comparison to other countries, and in-person services were allowed to resume while abiding by the imposed regulations. Furthermore, when COVID-19 restrictions were eased as the first wave of the pandemic was becoming less severe, in-person services were resumed gradually.

Several of the adaptations were influenced by the decision of organisations to prioritise services for the most vulnerable adolescents either by developing inclusive services or ones that were specifically tailored to these groups. In addition, a few organisations noted that they tried to specifically reach adolescents living in remote rural areas, informal urban areas, or other disadvantaged communities due to the anticipated impact of the pandemic on underserved groups of adolescents. They used remote or digital services, based on the context and adolescents’ preferences. Finally, many of the organisations added mental health services to their packages due to the anticipated burden laid on adolescents by the COVID-19 pandemic. These reasons prompted many organisations to provide different services concurrently, and in some instances, by using a combination of different adaptations.

Most of the organisations reported that they consulted with one or more groups of adolescent target users, service providers, partners, and other key informants to develop their adaptations.

Part of the adaptations that were developed in consultation with adolescents, was with adolescent volunteers or target audiences. For example, in Fiji, youth volunteers were involved in linking the organisation with adolescents and young people in their communities to learn about their needs and preferences, which influenced the design and planning of the outreach components used during the pandemic. In Namibia, the organisation based its SRH education activities on using online messaging services. However, when they consulted adolescents, they learned that it was costly and required an internet subscription; thus they reverted to peer-to-peer learning while enacting COVID-19 protective measures. In Argentina, the government conducted interviews with adolescent users to assess the barriers to access to services, the impact of COVID-19 on the most vulnerable groups, and the feasibility of proposed options. Based on these discussions, it prioritised the delivery of SGBV care and providing contraceptives in neighbourhoods that are far from the delivery centres. In addition, it provided the needed devices to several facilities to enable them to deliver telehealth consultations. On top of that, they utilised radio and television to disseminate SRH information to areas that lacked access to internet services. Finally, in different countries, when lockdown measures eased, and in-person services were resumed, many organisations maintained remote and digital services due to the preference of adolescent clients.

### Delivery and implementation of adaptations to SRH programmes

Many of the organisations were able to adapt their consultations and counselling services provided at healthcare facilities during the lockdown by abiding by COVID-19 guidelines and employing protective measures, using personal protective equipment (PPE), maintaining social distancing, having shorter consultation durations, and limiting the number of beneficiaries in waiting areas. Washing stations, sanitisers, soap, and masks were made available to the visitors, and appointment scheduling was used to avoid crowdedness. When transportation was restricted by government-mandated curfews, a few organisations were able to get exemptions for the mobility of their staff or adolescents accessing SRH services. When public transportation was limited, some organisations arranged for specific transportation for adolescents to be able to visit healthcare facilities. On the other hand, when adolescents did not prefer to have in-person contact, or no in-person services were running due to COVID-19-related mandated shutdowns, other approaches were used, such as telemedicine, phone calls, or WhatsApp calls, to communicate with service providers. In one of the case studies, dispensaries were used to provide condoms, and in two others, e-Pharmacies were used to allow adolescents to order SRH commodities. In areas where it was difficult for adolescents to reach healthcare facilities or did not have access to mobile phones or the internet, outreach teams were deployed to their communities, and healthcare providers distributed SRH commodities and provided consultations, counselling, and support through their mobile clinics or by conducting at-home visits. When it was not possible for organisations to resume their in-person services, they referred adolescents to other sites. This required the organisations to map available services to refer adolescents to a functioning healthcare service.

Organisations resorted to digital-based approaches to provide SRH information using websites, short messaging services (SMS), social media, and mHealth apps. Some organisations used social media platforms and apps such as Facebook, Zoom, and WhatsApp groups to carry out SRH education sessions. Furthermore, organisations adapted the way they promote the availability of services, whether it was through mass messaging with SMS, social media channels, and calls. Another approach was assisting adolescents in scheduling their appointments and coordinating their visits by providing adolescents with a WhatsApp to help with the scheduling of appointments and visits to the operating clinics.

In a few of the case studies, organisations either provided small incentives to adolescents to cover the cost of access to the internet, used social media apps that are available within the basic internet bundles, or included offline versions of their features. Other organisations tended to adolescents who may not have access to the internet or smartphones by using radio and television for edutainment programmes. As for services that were housed in the schools before the pandemic, they were mostly provided remotely. A few of the schools were able to resume their SRH education activities in small groups while employing COVID-19 protective measures when the lockdown measures were eased by their respective governments. Details of the adaptations used by the organisations appear in Table 2.

In the following section, we provide an overview of the different adaptations used to provide adolescents with SRH programmes and mental health services.

### Adaptations to SRH programmes

#### SRH education

Most organisations shifted to providing SRH education programmes using remote options, mostly with digital modalities. Organisations primarily used Facebook pages for asynchronous activities or WhatsApp for both synchronous sessions and asynchronous access to information. WhatsApp was used for carrying out sessions using WhatsApp group video calls, sharing educational audio and video messages and social media posts, and having a dedicated space where SRH educators could engage with young people and respond to their queries, whether individually or as a group. In addition, virtual meeting apps such as Zoom and Google Meet were commonly used for conducting online sessions and holding SRH talks or discussions with adolescents. Furthermore, organisations used websites or mobile apps to disseminate SRH information. For instance, Safeguard Young People (SYP) provided adolescents with information about reproductive health, including menstrual health, and gender-based violence on their “TuneMe” website, which was provided as part of the basic Internet bundle without any additional charges. Moreover, OneWorld Benin used its mobile app to provide SRH information to adolescents in a confidential manner. In addition to the digital tools, six organisations utilised radio broadcasting to remotely provide SRH information through dialogues, structured lessons, and edutainment programmes. In Myanmar, BBC Media Action supported the development of radio and digital content for adolescents with disabilities. Furthermore, Love Mater India’s radio weekly episodes provided adolescents with SRH information after the onset of the pandemic. On the other hand, some of the organisations were able to continue their in-person SRH education activities with some modifications. In Myanmar and India, organisations continued their face-to-face group sessions, but with reduced sizes to better observe social distancing measures.

Five organisations used alternative means to resume their CSE activities. These adaptations included the use of asynchronous online learning modules and synchronous online group sessions, in addition to social media and messaging apps. In India, a seven-module CSE programme was delivered via online sessions to both in-school and out-of-school adolescents. In the Philippines, online sessions were complemented by virtual groups and hotline services. In Zimbabwe, the Ministry of Education had the teachers adapt the CSE programme to reach adolescents through radio edutainment lessons. In Myanmar and Namibia, in-person activities were adapted to comply with COVID-19 prevention protocols. Moreover, messaging apps were also used to provide SRH information snippets that were adapted from the CSE curriculum.

#### Contraception services

Seven organisations from the DRC, India, Myanmar, Uganda, Namibia, Fiji, and Nigeria provided contraceptives in their clinics that operated in line with COVID-19 safety guidelines, with social distancing, use of masks, spacing between appointments, and operating in shifts convenient for clients. Two organisations, from the DRC and India, installed condom dispensers and boxes in accessible locations in health facilities for a “quick grab and go”. Additionally, one organisation set up kiosks that were installed near the outpatient departments of hospitals and were operated by female health workers. On the other hand, multiple organisations used outreach activities, mobile clinics, or household visits to provide contraceptives. For instance, two organisations from the DRC and the Philippines distributed contraceptives door-to-door. In Nigeria, contraceptives were provided upon request during household visits. Moreover, in Fiji and India, mobile clinics provided contraceptives, counselling, and referrals. In addition to providing contraceptives, four organisations provided contraception-related counselling services, either at their clinics, by outreach teams, or remotely through online or tele-counselling.

#### Abortion and post-abortion care

Three case studies were specific to abortion and post-abortion care. In Argentina, the Directorate of Adolescents and Youth and the Ministry of Health established a coordinated response to SRH issues through an intersectoral network of partners that covered the education, justice, and social development sectors. This network was able to refer requests for abortions from adolescents to the concerned service providers. Furthermore, they developed telehealth services guidelines and were able to provide remote consultations and counselling by phone or using telehealth arrangements. In the Philippines, one organisation assigned a hotline specifically for post-abortion care referrals, and provided post-abortion care in its clinics while complying with the COVID-19 safety protocols. Similarly, in India, health facilities of one organisation provided abortion and post-abortion care that was modified according to COVID-19 precautions. This was complemented by a community outreach component to promote the availability of the services and assist in the scheduling of appointments. Furthermore, it arranged for transportation using their ambulances to circumvent the mobility restrictions imposed by the lockdown.

#### Menstrual health

In India, Myanmar, and Nigeria, organisations distributed sanitary napkins or pads to adolescent girls at home. Among those, some prioritised adolescent girls with greater needs, such as those living in shelters, those with disabilities, and those living in poor households. Furthermore, they distributed other items such as face masks, sanitisers, and soaps, as well as iron-folic acid tablets and food packages.

In Nigeria, social media and radio edutainment programmes were used to provide information about menstrual health to adolescents. In Nepal, information was shared through online platforms and included lessons on how to make reusable sanitary napkins at home during the lockdown. In India, menstrual health education sessions were conducted with adolescent girls and boys via WhatsApp, and virtual activities such as poetry and mobile phone film-making competitions took place to engage adolescents and provide them with information.

#### SGBV care

Multiple case studies provided examples of adaptations to SGBV care. In Argentina, remote consultations and counselling were provided by phone or using telehealth arrangements when requested. In the Philippines, one organisation developed a Facebook-based platform to create a virtual community for adolescents where professionals respond to their SGBV concerns. In addition, it provided SGBV counselling and mental health support in its clinics and set up a hotline for SGBV care referrals. In Nigeria, the Stand With A Girl created a virtual safe space using WhatsApp groups to discuss SRH topics with adolescent girls, with a particular focus on SGBV, and the Stand To End Rape Initiative (STER) used phone services to support those who experienced SGBV and linked them to appropriate service providers. They provided mental health counselling via Zoom and Skype or used text messages for adolescents who preferred that option for communication. In addition, they arranged for transportation and facilitated the scheduling of services when needed.

#### Care for adolescents living with HIV

Four case studies provided responses that were particular to adolescents living with HIV. From Zimbabwe, Africaid and the Ministry of Health and Child Care, through the Zvandiri Programme, established a virtual case management system for adolescents living with HIV, built virtual support groups, provided virtual clinical and psychosocial HIV services, and shared reminders via their app to follow-up on adherence to treatment. Furthermore, their community adolescent treatment supporters initiated outreach visits, where they distributed ARTs, facilitated the measurement of the viral load, and provided the needed counselling and support. In India, online psychosocial support and tele-counselling were used to promote adherence to the ARTs. In the Philippines, HIV testing kits were distributed to adolescents. Finally, in Kenya, the University of Nairobi established virtual groups, through Zoom and Skype, to provide self-care, psychological first aid, and referral services, primarily for pregnant adolescents and adolescent parents living with HIV. In addition, they distributed ARTs door-to-door.

#### HPV vaccination

One case study reported on an adaptation to the national HPV vaccination programme In Laos. The vaccination programme was halted because of the closure of schools. The Ministry of Health shifted the programme setting to health centres and added mobile clinics in hard-to-reach sites. They sent messages through various channels to reach communities, parents, and girls and encouraged all eligible girls to visit the nearest centre or mobile clinic to receive the HPV vaccines.

### Distribution of SRH commodities

In two case studies from Uganda, UNFPA partnered with two for-profit organisations that provided app-based services. One of them was the e-Shop app, Jumia, which before this partnership did not provide a youth-centred SRH service. The other was SafeBoda, a motorcycle taxi app providing access to safe transportation. Both Ugandan organisations developed an e-Pharmacy section that could be accessed by adolescents to purchase SRH commodities and have them delivered with privacy and confidentiality, without additional delivery costs. Jumia increased its existing SRH products’ inventory, and SafeBoda onboarded ten major pharmacies to allow for the online ordering of SRH commodities. This allowed adolescents to order a range of commodities that included HIV testing kits, pregnancy testing kits, condoms, and menstrual health products, among others. Furthermore, with the support of UNFPA, Jumia had its customer service staff trained on SRH topics and provided information for their clients. On the other hand, Safeboda drivers delivered condoms to community health workers, peer educators, and village health teams to distribute them free of charge in their respective communities.

### Adaptations to mental health services

Many organisations, unprompted by WHO, included information about mental health services that they provided to adolescents during the pandemic, either as a complementary component of particular SRH services, such as HIV, SGBV, and abortion care, or as standalone services. For example, in India and Kenya, online counselling and psychosocial support were provided to adolescents living with HIV. In Zimbabwe, home visits with care teams provided adolescents with counselling and support. In Nigeria, mental health education, counselling, and psychosocial support were provided using a range of delivery mechanisms, such as phone services, virtual meeting apps, and text messaging. In Zimbabwe, call centres were used to refer those in need to appropriate services. Additionally, in Namibia, mental health support was provided through door-to-door outreach and mobile clinics.

### Monitoring of and follow-up on adaptations to SRH programmes

Organisations reported using a variety of methods and indicators to monitor their activities. The information was gathered through surveys, social media metrics, tracking sheets for service utilisation and commodities distributed, case management data, and feedback from users, healthcare providers, and staff across the different platforms. In-person data collection was impacted by the pandemic in most cases. Therefore, follow-up channels included phones, social media, virtual meeting apps, and messaging apps. In some cases, small group meetings took place to follow up on ongoing activities. Some unique examples provided real-time information for follow-up and monitoring of the adapted SRH services. For instance, e-Pharmacies provided real-time information about the number of clients and the number of SRH commodities that were purchased and delivered. Furthermore, the Zvandiri programme had its mobile app (ZVAMODA) for case management that was able to provide real-time data for follow-up on adolescents living with HIV registered on the app. They were able to track their HIV care needs and preferences and follow-up on their treatments and their adherence.

The most used indicators were “reach” and “utilisation” measures. For example, organisations measured the reach of and engagement with their social media and online resources, which included the number of views of webpages or social media posts, the number of unique online visitors, the number of resources downloaded, and the number of participants in their online sessions. Service utilisation measures included the number of adolescents who were provided with counselling, consultations, and commodities, in addition to the number of referrals through hotlines, messaging apps, or outreach activities.

Less than half of the organisations provided specific data at the time of collecting these case studies, although they mentioned the methods they were using or intended to use for data collection and follow-up. Among those that provided specific information, a few did not provide adolescent-specific data. The information shared by the organisations reported positively about the reach and coverage of their adapted services and the satisfaction of adolescent users. A summary of data provided in the case studies can be found in Table 3. At the time of this study, it was not possible to obtain information about the evaluation of these programmes.

**Table 3.** Outputs of the adapted adolescent SRH services during the COVID-19 Pandemic

Organisation	Data provided
Africaid (Zavandiri) and the Ministry of Health and Child Care – Zimbabwe	• Retained more than 45,000 children, adolescents and young people living with HIV who remained engaged in HIV care throughout the COVID-19 pandemic
Les Ailes Du Cœur-DRC	• Recruited 2,023 new users of modern contraceptive methods
	• Distributed 43,200 condoms
Beyond Beijing Committee (BBC) – Nepal	• Assisted 2000 women and girls through toll-free numbers during the lockdown period
	• Distributed reproductive health packages to 1389 women and girls
Directorate of Adolescents and Youth and the Ministry of Health- (DiAJu)- Argentina	• Regarding overall consultations referred to health workers, the proportion of adolescents referred increased from 33% to 45%
	• Face-to-face counselling constituted only (11%) as the virtual modality was the most preferred method (89%).
Family Planning Association of the Philippines (FPOP)	• Reached 400,000 people with a short series of online webinars
	• Communicated with 61,456 clients through YouRHotline
Footprints Foundation – South Africa	• Reached 14,573 girls across and donated 42,720 packs of sanitary pads in seven provinces
Love Matters India (LMI)	• Reached 10,000 young people through LMI WhatsApp service
	• Reached 500,000 young listeners through our radio programs
MAMTA Health Institute for Mother and Child- India	• Installed of 19 condom boxes across health facilities
	• Placed 14 kiosks were across facilities that reached up to 4763 couples
	• Involved 81 “Chugal Chachis” in positive gossiping about family planning, who reached over 6500 women across different sites
Marie Stopes International-Myanmar	• After implementing services adaptations, the client load has gradually increased back to around 6000 per month, from 1500 in the month of April 2020
Marie Stopes International-Uganda	• Adolescents continued to make up at least 20% of BlueStar clinic clients during the pandemic
Ministry of Health in Laos PDR	• Around 280,000 girls received the first dose of the HPV vaccination in Laos
	• The overall vaccine coverage by July 2020 was 77%
Namibia Planned Parenthood Association (NAPPA)	• Reached 649 young people Walvis Bay and provided them with information, and referrals
OneWorld Bénin	• Reached 1000 young persons
Society for Nutrition, Education and Health Action (SNEHA) – India	• Distributed 26,080 sanitary napkins to 1273 adolescent girls in this period
Safeguard Young People (SYP)/UNFPA ESARO	• The “TuneMe” website had of 11,866 page views
Talking About Reproductive and Sexual Health Issues (TARSHI) – India	• Self-care resources on their webpage have been accessed 2400 times, with 7000 page views, and were downloaded over 1200 times
UNFPA (Jumia) – Uganda	• Within the first month of launching the initiative in November 2020, it reached 38,500 people with SRH information through online promotion and responded to 2200 orders of SRH products
UNFPA and SafeBoda – Uganda	• Provided 815,325 people with information on SRH through various SafeBoda communication platforms, including social media, since its launch in June 2020
	• Distributed 1,175,040 free condoms in communities around Kampala city
	• On the online Personal Health Pharmacy, 3075 orders were made on the app, and 4720 items were delivered
Youth in Action (Y-ACT) – Kenya	• Provided 8725 women and girls with sanitary pads

## Discussion

This study set out to explore how organisations adapted their services to respond to the SRH needs of adolescents during the early phase of the COVID-19 pandemic. We gathered, analysed, and synthesised lessons learned from 36 case studies about the adaptations to service provision by 41 organisations from 16 LMICs, that provided SRH information and services to adolescents. The case studies covered face-to-face, remote, digital, and non-digital adaptations that were used to provide a wide range of SRH and mental health services, to different groups of adolescents. They provide useful lessons in terms of their development, implementation, and follow-up.

Our analysis showed that these adaptations covered a wide array of SRH services, mostly SRH education and access to contraception services. In addition, the study included examples of adaptations to services related to HIV care, menstrual health care, safe abortion and post-abortion care, SGBV care, distribution of SRH commodities, and a unique case study about adaptations used to provide HPV vaccination to adolescents. The case studies targeted a diverse group of adolescent groups, mostly adolescent girls, adolescent school students, or adolescents in nearby communities. A few of these efforts targeted other groups, including vulnerable adolescents, such as out-of-school adolescents, adolescents living with HIV, pregnant, parenting, or married adolescents, adolescents living in rural or urban informal settings, adolescents with disabilities, and LGBTQ+ adolescents. These examples showed that it was possible to adapt a range of SRH services in different contexts to reach adolescents, including those who are especially vulnerable, after the start of the pandemic. Furthermore, one of the positive findings is that adolescents’ mental health was prioritised by many organisations that provided them with mental health services either as a component of the SRH services or as a standalone component. Organisations cited the exacerbation in the burden on adolescents’ mental health due to the pandemic, as reported by multiple studies,^13,27^ as a reason for including mental health services as part of their adaptations.

The study provided insights regarding the development, implementation, and monitoring of adaptations to SRH services for adolescents. Firstly, many organisations developed their adaptations to SRH services as a direct response to the impact that lockdown measures, services’ closures, and mobility restrictions had on limiting or interrupting the access of adolescents to SRH services during the early stages of the COVID-19 pandemic.^8^ Furthermore, they developed their adaptations to reach underserved or vulnerable adolescents to mitigate the impact of the COVID-19 pandemic on these groups.^15^ As the adaptations were meant to circumvent the previously stated barriers resulting from the pandemic or the responses to it, they developed one or more adaptations to resume SRH services in health facilities and schools, implemented outreach activities to reach adolescents in their communities, referred them to other functioning SRH services when services were shut down, and used digital- and remote-based services when in-person static services were not possible or were not the option preferred by the adolescents. It is worth noting that some of the case studies provided examples of adaptations developed in consultations with adolescents, whether they were adolescents who contributed to service delivery or were part of the intended target audience. Consulting adolescents may have increased the chances of providing them with services that responded to their needs and preferences while taking into consideration factors such as the availability of transportation, cost of the internet, digital literacy, or access to smartphones, among other factors, in line with what has been reported elsewhere.^8^ Therefore, they arranged for transportation for adolescents, provided small incentives for internet use, or used low-cost approaches that used basic internet bundles, enabled cost-free access to offline versions of their approaches, and implemented outreach activities for those who did not have regular access to the internet and had limited mobility.

Secondly, in terms of the delivery and implementation of the adapted services, when service closure was not mandated or when COVID-19 restrictions were eased, in-person services were provided. Organisations modified their services in line with required COVID-19 safety measures by using PPE, maintaining social distancing, scheduling appointments, and spacing between these appointments. As a result, many organisations maintained their on-site services, but some of them also used remote approaches such as outreach teams, mobile clinics, community health workers, or young peers to provide SRH education, services, commodities, and referrals. There were a few examples of the provision of remote services using relatively innovative approaches, such as condom dispensers, e-Pharmacies, and no-contact delivery of SRH commodities, and radio and online SRH edutainment programmes. In addition, many organisations used helplines to provide counselling and referrals and to assist in the scheduling of appointments. Most remote services relied on digital adaptations, such as the use of social media, SMS, mobile apps, e-Pharmacy apps, virtual meeting apps, and online websites to provide SRH education or counselling, and assist in referrals, scheduling of appointments, and advertising for in-person services that were being resumed in the different clinics. Among the organisations that used digital adaptations in their SRH services, most used more than one modality of the previously mentioned adaptations. Additionally, most of the adaptations were simple rather than a complete revision of SRH programmes, in line with the objective of nimbly and quickly responding to the needs of adolescents in the early phase of the pandemic. As reported by Malkin and colleagues, simple and measured adaptations to existing services could be of promising value in terms of reaching adolescents and providing them with services with the needed urgency.^28^ Moreover, a considerable number of organisations used a combination of digital and face-to-face adaptations to the SRH services to achieve a synergetic effect. Even the organisations that relied on digital-based interventions at the beginning of the pandemic reported shifting their adaptations when the lockdown measures were eased to include more in-person components. Many organisations opted to provide in-person services in addition to their remote and digital-based services to maintain the preferred mode of delivery of SRH services by adolescent users or to ensure continuity of services as they were entering the second wave of the pandemic. According to a study in the Philippines, the use of both modalities – physical and virtual – to provide consultations and counselling to adolescents led to higher levels of utilisation by adolescents and young people when compared to using only one method.^22^

Finally, in terms of monitoring and follow-up, organisations resorted to the use of phones, social media, SMS, and virtual meeting apps to follow up on their operations and communicate with their teams. Most organisations provided information about the tools used for monitoring and follow-up. While less than half provided relevant data, the organisations that shared their information reported positively about the reach and coverage of their adapted services or the satisfaction of adolescent users. Still, this should raise concerns about the importance of monitoring and then evaluating these adaptations.

To put our study in context, we compare it to a number of examples of adaptations that were documented in the literature. Some examples described adaptations to in-person services, whether they were carried out in health facilities and schools or through outreach activities. In Brazil and Zimbabwe, social distance measures and infection control measures were observed in health facilities and included mandating social distancing, mask-wearing, providing PPE for health providers, providing some services outdoors, and installing handwashing facilities.^21,29^ In Indonesia, a teacher-led school-based CSE programme continued in classes – in some schools – with reduced student capacity and observing social distancing measures.^30^ As for outreach activities, there is an example from Kenya, where youth community health volunteers were mobilised in their communities to reach young adolescents and provide them with SRH information, counselling, and commodities such as condoms and contraceptive pills.^28^ Similar to our study, while in-person activities had their value in providing adolescents with face-to-face support,^21,31^ it was important during the early phase of the pandemic to provide alternative options for adolescents for whom access was difficult and to respond to their preferences in terms of the approach used to deliver the service. For instance, in India and Brazil, telemedicine was used to provide family planning counselling^32^ and HIV care^29^ to adolescent girls and adolescents living with HIV, respectively. As for SRH education, in-person SRH education lessons were digitised and disseminated to adolescent girls through WhatsApp in Nigeria,^28^ and social media were utilised to provide HIV prevention information to adolescents in Zambia.^33^ These examples document different adaptations to SRH education, in addition to contraception services and HIV care, with similar strategies to the ones reported in our case studies. Yet, our set of case studies could be of added value to the base of evidence as it contains information about adaptations to a wider range of SRH services that includes safe abortion and post-abortion care, SGBV care, HPV vaccination, and examples of the distribution of a variety of SRH commodities, in addition to mental health services, that were provided to diverse groups of adolescents, sometimes using different approaches, concurrently, to provide different services and increase their reach.

### Study implications

Our study findings show that organisations in the LMICs nimbly adapted a wide array of SRH services that were provided to different groups of adolescents in different contexts, using multiple modalities, including physical and remote services, with digital and non-digital adaptations, sometimes as standalone adaptations and at other times in combination, to reach the different groups of adolescents, and ensure continuity of services. In addition, the study has shown that mental health issues were prioritised while addressing SRH services; this could well provide an opportunity for exploring future linkages between mental health and SRH programming for adolescents. Furthermore, the study pointed to lessons learned from adapting SRH services according to the context of the early phase of the pandemic, the level of severity of the first wave, the extent of lockdown measures, and governments’ responses. In addition, in some instances, adaptations benefited from the involvement of adolescents in the design and implementation of these adaptations, as their input facilitated reaching the best iterations of these adaptations to enhance chances of reaching adolescents and responding to their SRH needs.

Some of these modalities were in use prior to the pandemic,^34,35^ yet these case studies show that there has been a clear expansion in using the different adaptations, including digital adaptations, during the pandemic. Further, information provided on such adaptations gives an insight into their perceived benefits, despite the extenuating circumstances in which they were developed and implemented. Rigorous evaluations are needed to determine if these adaptations could influence adolescent SRH outcomes in the long term. For instance, in Zambia, a study reported that a combination of in-person and digital adaptations to HIV testing services was safe, feasible, and resulted in more uptake of HIV testing services among adolescents.^33^ On the other hand, in Zimbabwe, a study reported that some of the adaptations used to provide SRH services had a negative impact on reaching adolescents, their retention, and the acceptability of the adapted services, as core elements of the services were not optimally adapted, which affected the quality of services delivered by the healthcare providers.^21^ Therefore, careful evaluation of these adaptations should be considered, particularly for longer-term outcomes.

While WHO has declared that the COVID-19 pandemic is no longer a public health emergency,^36^ lessons learned from this study can feed into discussions about the positioning of these adaptations into preparedness programmes to mitigate the impact of future public health emergencies on adolescents’ access to SRH services. It could be harder to apply the lessons learned in areas of humanitarian crises resulting from conflict or natural disasters where infrastructure is damaged, population displacement occurs, and internet communication is restricted, but other emergencies could benefit from considering the different examples where small-scale adaptations were rapidly made and implemented with the available resources in the face of the pandemic. Therefore, it is important to take into account the context when considering the set of adaptations presented in our study. Future research could explore if these adaptations were utilised in the following waves of the pandemic, particularly when COVID-19 vaccinations started to roll out the following year, albeit in some countries more than others,^37,38^ or in the post-pandemic era, and if that is informed by implementation evidence, as it could draw lessons on how these adaptations could be situated in current ASRH programming.

### Limitations

The study has a few limitations. Firstly, most of the case studies came from Africa and Asia. While the call had no geographical restrictions and all regional offices were approached, responses came mainly from these regions. This reaffirms the opportunistic nature of the study, and while Africa and Asia constitute the places where most adolescents are currently living globally,^39^ the study could have benefited from better representation. Secondly, only a few of the case studies targeted some of the most vulnerable groups of adolescents. Some adolescent groups, such as LGBTQ+ adolescents, adolescents living with disabilities, adolescents living in rural or informal urban areas, and pregnant adolescents or adolescent mothers, were not highlighted in enough depth in this study. In the future, targeted research should be carried out to learn about how programme adaptations could respond to the special needs of these groups to ensure that they are not left behind. Thirdly, there were some issues with the quality of data and information reported by the organisations. While the information provides insights in terms of the potential feasibility and acceptability of the adaptations made, as reported by some of the organisations, it is crucial to point out that these results were not validated or verified. In the future, rigorous evaluation is needed to best attain a picture of the effectiveness of those interventions in achieving favourable results for long-term ASRH outcomes among adolescents. Finally, the findings of the study are particular to the early phase of the pandemic, particularly during or after its first wave, and with no additional information, it is important not to extrapolate the findings to the entirety of the pandemic, where different regulations were imposed, or COVID-19 vaccination may have rolled out. It is important to consider the context of these adaptations as rapid and nimble responses by the organisations that may have changed with time.

## Conclusion

The study showed that organisations operating in different countries and contexts were able to provide SRH and mental health services to adolescents despite the extenuating circumstances prevailing in the early phase of the COVID-19 pandemic. These adaptations were reported to be doable by programmers and acceptable to the targeted adolescents. Simple adaptations to existing programmes, the use of combinations of digital and non-digital services, and the involvement of adolescents in the design and implementation of these adaptations helped facilitate these adaptations. Lessons learned from this study could be extrapolated into future public health emergencies, but future research should curate rigorous evaluations to assess the effectiveness of these adaptations in achieving favourable results for long-term SRH outcomes among adolescents and focus on reaching the most vulnerable.

## Footnotes

* According to UNFPA, SRH commodities refer to SRH supplies, equipment, and kits that are core components of family planning, maternal and neonatal care, and STIs and HIV-related services. For example, they include contraceptives, family planning devices, pregnancy testing kits, delivery kits for use at home, midwife professional kits, condoms, and self-testing kits, among others.^9^

† According to UNESCO, Comprehensive sexuality education (CSE) is “a curriculum-based process of teaching and learning about the cognitive, emotional, physical, and social aspects of sexuality. It aims to equip children and young people with knowledge, skills, attitudes, and values that will empower them to: realize their health, well-being, and dignity; develop respectful social and sexual relationships; consider how their choices affect their own well-being and that of others; and understand and ensure the protection of their rights throughout their lives”.^26^ In different countries, these curricula or programmes may be adapted and referred to with different names.^26^

## References

[1] United Nations. UN DESA policy brief no. 140: a world of 8 billion [Internet]. 2022. Available from: https://www.un.org/development/desa/dpad/publication/un-desa-policy-brief-no-140-a-world-of-8-billion/.

[2] Chandra-Mouli V, Ferguson BJ, Plesons M, et al. The political, research, programmatic, and social responses to adolescent sexual and reproductive health and rights in the 25 years since the international conference on population and development. J Adolesc Health. 2019;65(6):S16–S40. doi:10.1016/j.jadohealth.2019.09.011 31761001

[3] Chandra-Mouli V, Akwara E, Engel D, et al. Progress in adolescent sexual and reproductive health and rights globally between 1990 and 2016: what progress has been made, what contributed to this, and what are the implications for the future? Sex Reprod Health Matters. 2020;28(1):1741495. doi:10.1080/26410397.2020.1741495 32254004 PMC7888102

[4] Jennings L, George AS, Jacobs T, et al. A forgotten group during humanitarian crises: a systematic review of sexual and reproductive health interventions for young people including adolescents in humanitarian settings. Confl Health. 2019;13(1):57. doi:10.1186/s13031-019-0240-y 31788022 PMC6880589

[5] Murewanhema G. Adolescent girls, a forgotten population in resource-limited settings in the COVID-19 pandemic: implications for sexual and reproductive health outcomes. Pan Afr Med J. 2020;37(Suppl 1):41. 10.11604/pamj.supp.2020.37.1.26970 PMC7846263 33552369

[6] Ng’andu M, Mesic A, Pry J, et al. Sexual and reproductive health services during outbreaks, epidemics, and pandemics in sub-Saharan Africa: a literature scoping review. Syst Rev. 2022 Aug 9;11(1):161. doi:10.1186/s13643-022-02035-x PMC9361234 35945580

[7] Goga A, Bekker LG, Van de Perre P, et al. Centring adolescent girls and young women in the HIV and COVID-19 responses. Lancet Lond Engl. 2020;396(10266):1864–1866. doi:10.1016/S0140-6736(20)32552-6 33271130

[8] Meherali S, Adewale B, Ali S, et al. Impact of the COVID-19 Pandemic on Adolescents' Sexual and Reproductive Health in Low- and Middle-Income Countries. Int J Environ Res Public Health. 2021 Dec 15;18(24):13221. doi:10.3390/ijerph182413221 PMC8701118 34948829

[9] UNFPA. Reproductive health essentials. Securing the supply: global strategy for reproductive health commodity security [Internet]; 2002. Available from: https://www.unfpa.org/sites/default/files/resource-pdf/securingsupply_eng.pdf.

[10] Groenewald C, Isaacs N, Isaacs D. Adolescent sexual and reproductive health during the COVID-19 pandemic: a mini review. Front Reprod Health. 2022 Mar 30;4:794477. doi:10.3389/frph.2022.794477 PMC9580774 36303613

[11] Mukherjee TI, Khan AG, Dasgupta A, et al. Reproductive justice in the time of COVID-19: a systematic review of the indirect impacts of COVID-19 on sexual and reproductive health. Reprod Health. 2021 Dec 20;18(1):252. doi:10.1186/s12978-021-01286-6 PMC8686348 34930318

[12] VanBenschoten H, Kuganantham H, Larsson EC, et al. Impact of the COVID-19 pandemic on access to and utilisation of services for sexual and reproductive health: a scoping review. BMJ Glob Health. 2022; Oct7(10):e009594. doi:10.1136/bmjgh-2022-009594 PMC9539651 36202429

[13] Ramaiya A, Chandra-Mouli V, Both R, et al. Assessing the health, social, educational and economic impact of the COVID-19 pandemic on adolescents in low-and middle-income countries: a rapid review of the literature. Sex Reprod Health Matters. 2023;31(1):2187170. doi:10.1080/26410397.2023.2187170 36987980 PMC10062253

[14] Singh L, Abbas SM, Roberts B, et al. A systematic review of the indirect impacts of COVID-19 on sexual and reproductive health services and outcomes in humanitarian settings. BMJ Glob Health. 2023 Nov;8(11):e013477. doi:10.1136/bmjgh-2023-013477 PMC10660896 37984894

[15] Lindberg LD, Bell DL, Kantor LM. The sexual and reproductive health of adolescents and young adults during the COVID-19 pandemic. Perspect Sex Reprod Health. 2020;52(2):75–79. doi:10.1363/psrh.12151 32537858 PMC7323157

[16] Tolu LB, Feyissa GT, Jeldu WG. Guidelines and best practice recommendations on reproductive health services provision amid COVID-19 pandemic: scoping review. BMC Public Health. 2021;21(1):276. 10.1186/s12889-021-10346-2 PMC7856605 33536001

[17] World Health Organization. Continuing essential sexual reproductive, maternal, neonatal, child and adolescent health services during COVID-19 pandemic: practical considerations [Internet]. WorldHealth Organization Regional Office for South-East Asia; 2020. Available from: https://apps.who.int/iris/handle/10665/332162.

[18] World Health Organization. COVID-19: operational guidance for maintaining essential health services during an outbreak: interim guidance, 25 March 2020. WorldHealth Organization [Internet]; 2020. Available from: https://apps.who.int/iris/handle/10665/331561.

[19] UNFPA. Responding to the sexual and reproductive health needs of adolescents during the COVID-19 crisis [Internet]; 2020. Available from: https://www.unfpa.org/resources/responding-sexual-and-reproductive-health-needs-adolescents-during-covid-19-crisis.

[20] Duby Z, Bunce B, Fowler C, et al. Adaptation and resilience: lessons learned from implementing a combination health and education intervention for adolescent girls and young women in South Africa during the COVID-19 pandemic. Front Health Serv. 2022 Jun 3;2:903583. doi:10.3389/frhs.2022.903583 PMC10012768 36925833

[21] Mackworth-Young CRS, Mavodza C, Nyamwanza R, et al. “Other risks don’t stop”: adapting a youth sexual and reproductive health intervention in Zimbabwe during COVID-19. Sex Reprod Health Matters. 2022;30(1):2029338. doi:10.1080/26410397.2022.2029338 35192449 PMC8865116

[22] Okunogbe A, Meekins M, Saalim K, et al. Utilization of adolescent health services during the COVID-19 pandemic: evidence on impact and adaptations from a rapid assessment survey in the Philippines. BMC Public Health. 2023;23(1):493. doi:10.1186/s12889-023-15102-2 36918863 PMC10013233

[23] Rothengatter W, Zhang J, Hayashi Y, et al. Pandemic waves and the time after Covid-19 – consequences for the transport sector. Transp Policy. 2021;110:225–237. doi:10.1016/j.tranpol.2021.06.003 PMC8481202 34608362

[24] Kwok KO, Huang Y, Tsoi MTF, et al. Epidemiology, clinical spectrum, viral kinetics and impact of COVID-19 in the Asia-pacific region. Respirol Carlton Vic. 2021;26(4):322–333. doi:10.1111/resp.14026 PMC8207122 33690946

[25] Salyer SJ, Maeda J, Sembuche S, et al. The first and second waves of the COVID-19 pandemic in Africa: a cross-sectional study. Lancet Lond Engl. 2021;397(10281):1265–1275. doi:10.1016/S0140-6736(21)00632-2 PMC8046510 33773118

[26] UNESCO. Comprehensive sexuality education: for healthy, informed and empowered learners [Internet]; 2017. Available from: https://www.unesco.org/en/health-education/cse.

[27] Harrison L, Carducci B, Klein JD, Bhutta ZA. Indirect effects of COVID-19 on child and adolescent mental health: an overview of systematic reviews. BMJ Glob Health. 2022 Dec;7(12):e010713. doi:10.1136/bmjgh-2022-010713 PMC9808753 36585030

[28] Malkin M, Mickler AK, Ajibade TO, et al. Adapting high impact practices in family planning during the COVID-19 pandemic: experiences from Kenya, Nigeria, and Zimbabwe. Glob Health Sci Pract. 2022;10(4):e2200064. doi:10.9745/GHSP-D-22-00064 36041833 PMC9426984

[29] Dourado I, Magno L, Soares F, et al. Adapting to the COVID-19 pandemic: continuing HIV prevention services for adolescents through telemonitoring, Brazil. AIDS Behav. 2020;24:1994–1999. doi:10.1007/s10461-020-02927-w 32440973 PMC7241065

[30] Pinandari AW, Kågesten AE, Li M, et al. Short-term effects of a school-based comprehensive sexuality education intervention among very young adolescents in three urban Indonesian settings: a quasi-experimental study. J Adolesc Health. 2023;73(1):S21–S32. doi:10.1016/j.jadohealth.2023.01.030 37330818

[31] Mseke EP, Jessup B, Barnett T. A systematic review of the preferences of rural and remote youth for mental health service access: telehealth versus face-to-face consultation. Aust J Rural Health. 2023;31(3):346–360. doi:10.1111/ajr.12961 36606417

[32] Banke-Thomas A, Yaya S. Looking ahead in the COVID-19 pandemic: emerging lessons learned for sexual and reproductive health services in low- and middle-income countries. Reprod Health. 2021 Dec 14;18(1):248. doi:10.1186/s12978-021-01307-4 PMC8670615 34906177

[33] Phiri MM, Hensen B, Schaap A, et al. Adapting community-based sexual and reproductive health services for adolescents and young people aged 15-24 years in response to COVID-19 in Lusaka, Zambia: the implications on the uptake of HIV testing services. BMC Health Serv Res. 2022;22(1):503. doi:10.1186/s12913-022-07878-7 35421966 PMC9008386

[34] Huang KY, Kumar M, Cheng S, et al. Applying technology to promote sexual and reproductive health and prevent gender based violence for adolescents in low and middle-income countries: digital health strategies synthesis from an umbrella review. BMC Health Serv Res. 2022 Nov 19;22(1):1373. doi:10.1186/s12913-022-08673-0 PMC9675248 36401323

[35] Rea S, Zynda A, Allison B, et al. Adolescent perceptions of technology-based sexual and reproductive health services: a systematic review. J Adolesc Health Off Publ Soc Adolesc Med. 2022;71(5):533–544. doi:10.1016/j.jadohealth.2022.05.012 35717326

[36] World Health Organization. WHO director-general’s opening remarks at the media briefing on COVID-19—11 March 2020 [Internet]; 2023. Available from: https://www.who.int/director-general/speeches/detail/who-director-general-s-opening-remarks-at-the-media-briefing-on-covid-19—11-march-2020.

[37] Mathieu E, Ritchie H, Ortiz-Ospina E, et al. A global database of COVID-19 vaccinations. Nat Hum Behav. 2021;5(7):947–953. doi:10.1038/s41562-021-01122-8 33972767

[38] Watson OJ, Barnsley G, Toor J, et al. Global impact of the first year of COVID-19 vaccination: a mathematical modelling study. Lancet Infect Dis. 2022;22(9):1293–1302. doi:10.1016/S1473-3099(22)00320-6 35753318 PMC9225255

[39] UNFPA. Adolescent and youth demographics: a brief overview [Internet]. Available from: https://www.unfpa.org/sites/default/files/resourcepdf/One%20pager%20on%20youth%20demographics%20GF.pdf.

